# Supplementary material for: Protocol for Enrichment of Murine Cardiac Junctional Sarcoplasmic Reticulum Vesicles for Mass Spectrometry Analysis
Source: Int J Mol Sci. 2025 Sep 4;26(17):8602. doi: 10.3390/ijms26178602 (PMC12429420; doi:10.3390/ijms26178602)
Supplement: Supplementary file 1 [file ijms-26-08602-s001.zip › ijms-3796180-supplementary.pdf]

Supplementary Material

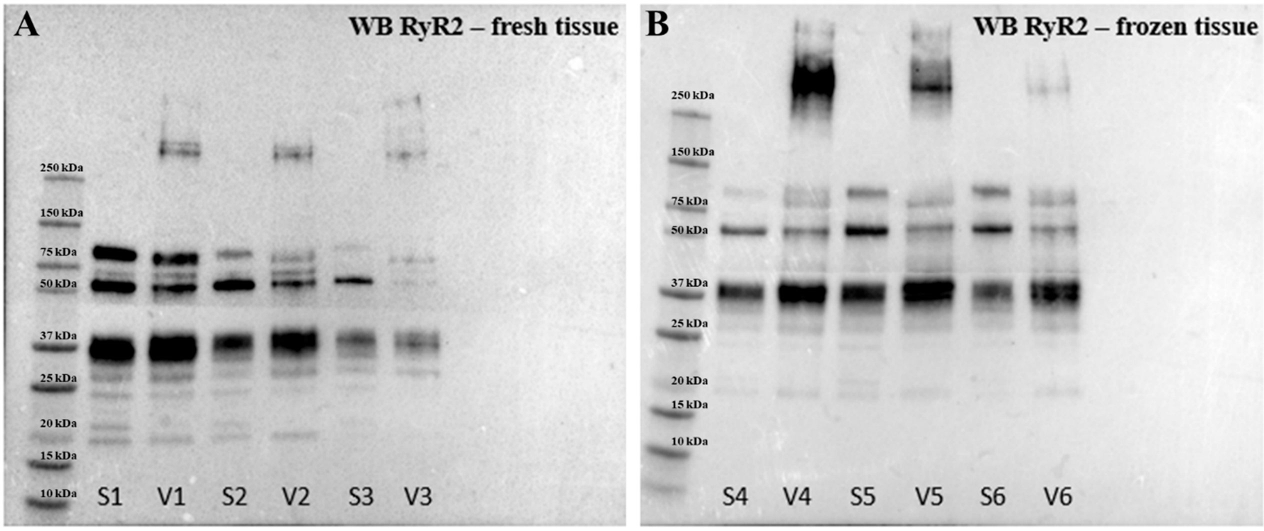

Figure S1: A) Anti RyR2 Western blot of 3 fresh hearts treated independently and simultaneously lysed with identical procedures. **B)** WB analysis of RyR2 of 3 mice hearts differently homogenized with Minilys beads beater. Prolonged bead beating leads to increased fragmentation of RyR2, rather than to an improved yield in the final vesicle preparation.

Table S1: List of the MS-identified proteins with endoplasmic and sarcoplasmic reticulum cellular localization, within the SR vesicles samples (V). Peptides: the maximal number of peptides identified from all included searches for the master protein of the protein group. Unique peptides: the number of unique peptide sequences identified from all included searches for the master protein of the protein group.

UniProt ID	Protein name	Peptides	Unique Peptides
Q9D517	1-acyl-sn-glycerol-3-phosphate acyltransferase gamma	9	9
P50172	11-beta-hydroxysteroid dehydrogenase 1	3	3
Q497N1	40S ribosomal protein S26	3	3
Q4VWZ5	Acyl-CoA-binding protein	2	2
B1ATI0	Aldehyde dehydrogenase	2	2
Q8BSY0	Aspartyl/asparaginyl beta-hydroxylase	8	7
Q8VCM8	BOS complex subunit NCLN	3	3
Q6GQT9	BOS complex subunit NOMO1	7	7
B9EHY2	CAAX prenyl protease	3	3
A0A0A6YVS2	Calcium load-activated calcium channel	2	2
Q8K1N1	Calcium-independent phospholipase A2-gamma	4	4
Q3TXE5	Calnexin	15	15
B9EHC7	Calsequestrin	10	10
P23953	Carboxylesterase 1C	6	2
Q8VCT4	Carboxylesterase 1D	17	14
P47934	Carnitine O-acetyltransferase	18	18

Q9D1I2	Caspase recruitment domain-containing protein 19	2	2
Q9CQB5	CDGSH iron-sulfur domain-containing protein 2	6	6
Q8VDP6	CDP-diacylglycerol--inositol 3-phosphatidyltransferase	2	2
Q544Z9	Cytochrome b5	5	5
Q8BMK4	Cytoskeleton-associated protein 4	17	17
Q3TVU9	Dehydrogenase/reductase SDR family member 1	6	6
Q99J47	Dehydrogenase/reductase SDR family member 7B	4	4
Q8CHS7	Dehydrogenase/reductase SDR family member 7C	3	3
O54734	Dolichyl-diphosphooligosaccharide--protein glycosyltransferase 48 kDa subunit	8	8
Q8BMR3	Dolichyl-diphosphooligosaccharide--protein glycosyltransferase subunit 1	15	15
Q3U505	Dolichyl-diphosphooligosaccharide--protein glycosyltransferase subunit 2	11	11
P61804	Dolichyl-diphosphooligosaccharide--protein glycosyltransferase subunit DAD1	2	2
A0A0R4J0D3	dolichyl-diphosphooligosaccharide--protein glycotransferase	2	2
Q3TL33	EF-hand domain-containing protein	4	4
P62631	Elongation factor 1-alpha 2	15	7
P20029	Endoplasmic reticulum chaperone BiP	28	25
P08113	Endoplasmin	19	18
A1L2Z3	ER membrane protein complex subunit 1	12	12
Q6A0D1	ER membrane protein complex subunit 2	3	3
Q99KI3	ER membrane protein complex subunit 3	3	3
Q8BFZ9	Erlin-2	4	4
Q9EQ06	Estradiol 17-beta-dehydrogenase 11	5	5
Q91V79	Fat storage-inducing transmembrane protein 1	2	2
Q3TFU8	GOLD domain-containing protein	2	2
O08795-2	Isoform 2 of Glucosidase 2 subunit beta	5	5
O70622-2	Isoform 2 of Reticulon-2	5	5
Q7TQ48-1	Isoform 2 of Sarcalumenin	26	26
Q9ET78	Junctophilin-2	15	15
D3Z041	Long-chain-fatty-acid--CoA ligase	41	37
Q8R1X1	Long-chain-fatty-acid--CoA ligase	3	2
Q3UN02	Lysocardiolipin acyltransferase 1	5	5
Q91V01	Lysophospholipid acyltransferase 5	3	3
Q6ZQI3	Malectin	4	4
E9QJW0	Microsomal glutathione S-transferase 1	2	2
Q3TPU5	MPN domain-containing protein	2	2
F2Z456	NADH-cytochrome b5 reductase	7	7
P37040	NADPH--cytochrome P450 reductase	9	9
Q9D024	PAT complex subunit CCDC47	3	3
Q9DCY1	Peptidyl-prolyl cis-trans isomerase	7	7
Q8CGN5	Perilipin-1	4	4

Q99L43	Phosphatidate cytidylyltransferase 2	6	6
Q8R5J9	PRA1 family protein 3	2	2
Q9WV91	Prostaglandin F2 receptor negative regulator	2	2
P09103	Protein disulfide-isomerase	17	17
P27773	Protein disulfide-isomerase A3	16	16
Q3THH1	Protein disulfide-isomerase A6	5	5
Q5XKN4	Protein jagunal homolog 1	2	2
Q80WJ7	Protein LYRIC	3	3
P61620	Protein transport protein Sec61 subunit alpha isoform 1	2	2
Q9CQS8	Protein transport protein Sec61 subunit beta	2	2
Q9D1G3	Protein-cysteine N-palmitoyltransferase HHAT-like protein	8	8
G3X8R0	Receptor expression-enhancing protein	6	6
Q9ES97	Reticulon-3	2	2
E9Q401	Ryanodine receptor 2	87	83
P19324	Serpin H1	5	5
Q6PET3	Signal peptidase complex catalytic subunit SEC11	2	2
A0A140LHG8	Signal peptidase complex subunit 2	3	3
P47758	Signal recognition particle receptor subunit beta	3	3
Q9CZI5	Small ribosomal subunit protein uS12	5	5
A2AL78	submitted name: Aspartate-beta-hydroxylase	3	2
Q3U7E6	Surfeit locus protein 4	3	3
Q8VBT0	Thioredoxin-related transmembrane protein 1	3	3
Q9D710	Thioredoxin-related transmembrane protein 2	5	5
Q3U9K9	Trafficking protein particle complex subunit	2	2
A0A286YCG8	Translocon-associated protein subunit alpha	3	3
Q9D8L3	Translocon-associated protein subunit delta	2	2
Q9D1D4	Transmembrane emp24 domain-containing protein 10	4	4
Q8R1V4	Transmembrane emp24 domain-containing protein 4	3	3
Q3TMP8	Trimeric intracellular cation channel type A	3	3
Q6P5E4	UDP-glucose:glycoprotein glucosyltransferase 1	6	6
Q99K23	Ufm1-specific protease 2	2	2
Q8K2C9	Very-long-chain (3R)-3-hydroxyacyl-CoA dehydratase 3	3	3
A0A5F8MQC8	very-long-chain enoyl-CoA reductase	6	6
Q6QD59	Vesicle transport protein SEC20	3	3
Q9WV55	Vesicle-associated membrane protein-associated protein A	4	3
O08547	Vesicle-trafficking protein SEC22b	2	2

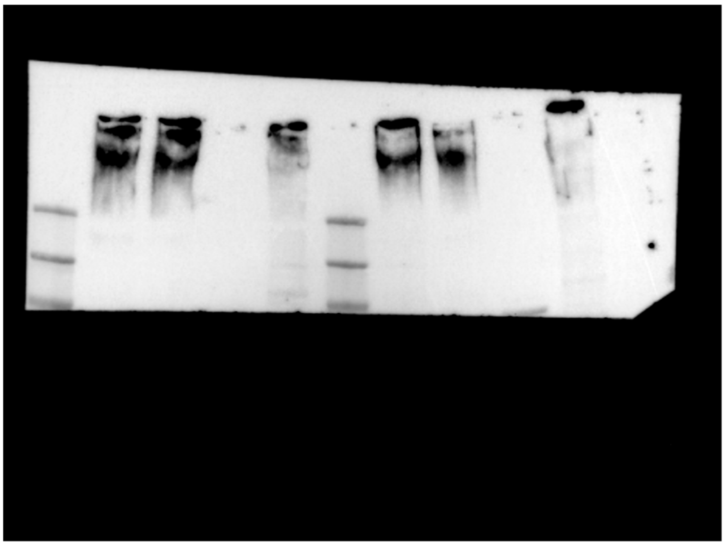

Figure S2: Raw image of the western blot for RyR2 corresponding to the experiment shown in Figure 2.

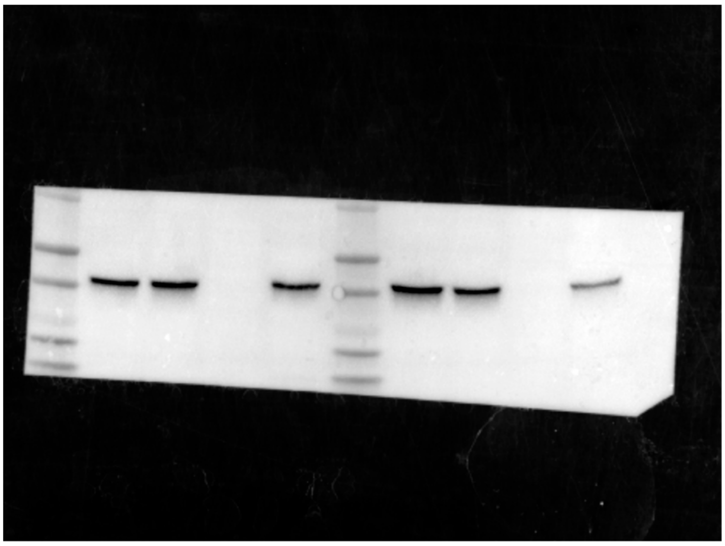

Figure S3: Raw image of the western blot for TRDN corresponding to the experiment shown in Figure 2.

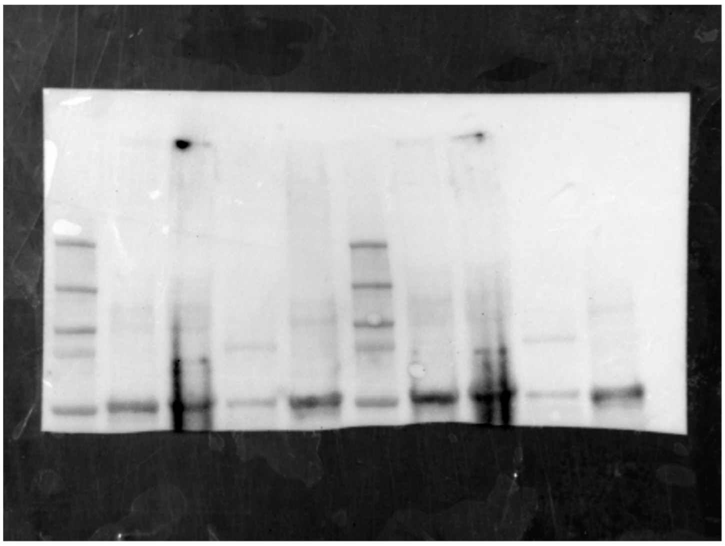

Figure S4: Raw image of the western blot for CASQ2 corresponding to the experiment shown in Figure 2.

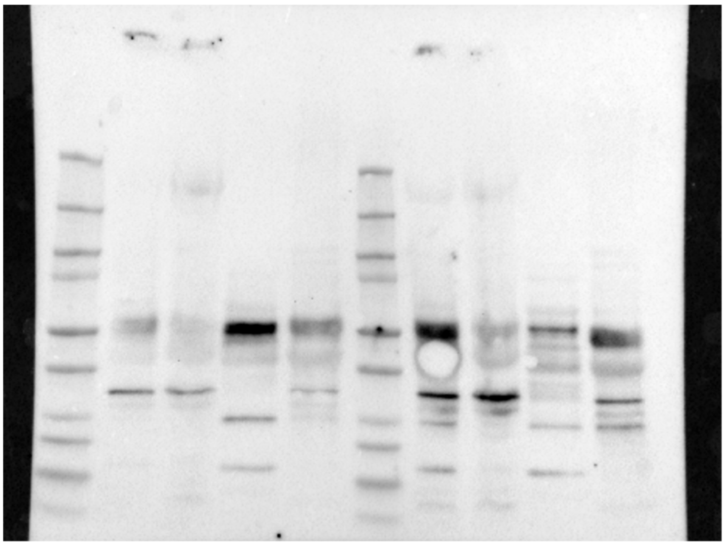

Figure S5: Raw image of the western blot for MCU corresponding to the experiment shown in Figure 2.

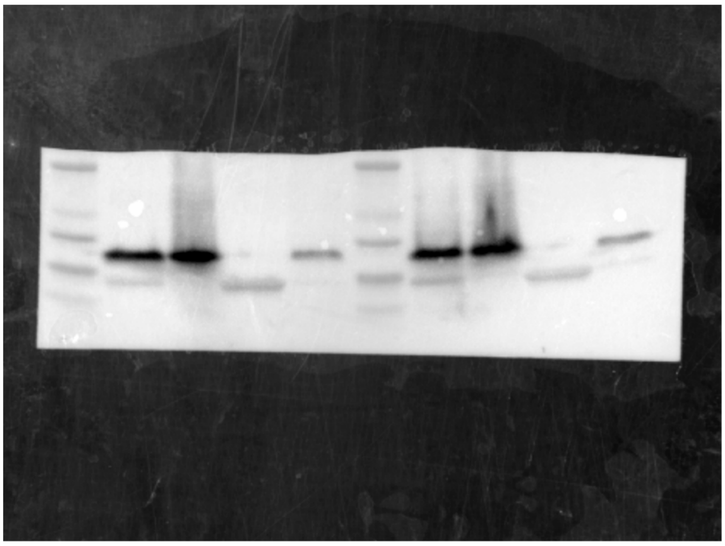

Figure S6: Raw image of the western blot for H3 corresponding to the experiment shown in Figure 2.
